# Entropy Generation of Carbon Nanotubes Flow in a Rotating Channel with Hall and Ion-Slip Effect Using Effective Thermal Conductivity Model

**DOI:** 10.3390/e21010052

**Published:** 2019-01-10

**Authors:** Nosheen Feroz, Zahir Shah, Saeed Islam, Ebraheem O. Alzahrani, Waris Khan

**Affiliations:** 1 Department of Mathematics, Abdul Wali Khan University, Mardan, Khyber, Pakhtunkhwa 23200, Pakistan; 2 Department of Mathematics, Faculty of Science, King Abdulaziz University, P.O. Box 80203, Jeddah 21589, Saudi Arabia; 3 Department of Mathematics, Kohat University of Science and technology, Kohat 26000, Pakistan

**Keywords:** Hall and ion-slip effect, entropy generation, CNTs, MHD, rotating system, thermal radiation, HAM

## Abstract

This article examines the entropy analysis of magnetohydrodynamic (MHD) nanofluid flow of single and multiwall carbon nanotubes between two rotating parallel plates. The nanofluid flow is taken under the existence of Hall current and ion-slip effect. Carbon nanotubes (CNTs) are highly proficient heat transmission agents with bordering entropy generation and, thus, are considered to be a capable cooling medium. Entropy generation and Hall effect are mainly focused upon in this work. Using the appropriate similarity transformation, the central partial differential equations are changed to a system of ordinary differential equations, and an optimal approach is used for solution purposes. The resultant non-dimensional physical parameter appear in the velocity and temperature fields discussed using graphs. Also, the effect of skin fraction coefficient and Nusselt number of enclosed physical parameters are discussed using tables. It is observed that increased values of magnetic and ion-slip parameters reduce the velocity of the nanofluids and increase entropy generation. The results reveal that considering higher magnetic forces results in greater conduction mechanism.

## 1. Introduction

Carbon nanotubes (CNTs) nanofluids have been widely used in a number of engineering applications. The current advances in technology have led to evolved integrated circuits, and the applications of these circuits are growing day by day. The extremely active field of nanomaterials research is supported by this progress. After the discovery of fullerene in 1985, nanoparticle structures, which are based on carbon, have fascinated and attracted the attention of the methodical research community to apply and understand their future. Xiao et al. [1,2] have investigated the absorptivity of nanofibers with capillary pressure impact using a fractal-Monte Carlo procedure, with the effect of thermal diffusion and thermal conduction. Xiao et al. [3] investigated the water flow over unsaturated permeable rocks. Liang et al. [4] studied an investigative model for the oblique absorptivity of gas transmission layer with electrical effects. Long et al. [5,6] studied the properties of puncture destruction on applied hydraulic-fracturing requests. Kroto et al. [7] deliberated that the relation of allotropic forms and carbon elements which can be selected in various hybridization forms; in this case, the structure of CNTs are one-dimensional, which has been examined thoroughly, because of their prospective application in nanoelectronics (Lijima [8]). A single sheet of graphene (a hexagonal lattice of carbon nanomaterial) can construct CNTs which are rolled onto a cylinder. Graphene, which has been extensively probed, is a carbon nanostructure in two dimensions [9,10]. However, CNTs show exceptional variations in their structure with regard to graphene because of the curvature [11]. Currently, the consideration of several investigators is to analyze nanotechnology which was created by Choi [12] in the year 1995. Nanofluid is one of the predominant units of nanotechnology, and it is a potential heat transference liquid. CNTs nanofluids have various uses, in structural (concrete, body arms, bridges, textiles, polyethylene, sports equipment, fire protection, flywheel), chemical (chemical nanowires, water filter, sensors, air pollution), mechanical (waterproof, oscillator), and electromagnetic (electromagnetic antennas, magnets, solar cells, buckypaper, light bulb filaments) materials, as transistors, and in cancer treatment, electrical circuits, optics, and many more. Investigators are focusing on CNTs because of such applications. Kang et al. [13] tested, on an experimental basis, and revised the precision of the results of Haq et al. [14] studied single wall and multiwall carbon nanotubes, taking water as a base fluid. Liu et al. [15] have determined that the combination of ethylene glycol and CNTs has greater conductivity than ethylene glycol devoid of CNTs. This review was done on ethylene glycol and synthetic engine oil in the occurrence of MWCNTs. A radiative Darcy–Forchheimer flowing of carbon nanotube with microstructure and inertia features was examined by Shah et al. [16] in recent times. The central structure block of nanoparticles is nanofluids. In the advancement of nanotechnology, the investigators, paid particular attention to this area. The understanding of heat diffusion in a magnetohydrodynamic nanofluid stream concluded that various geometrics are vital for transpiration, heat transfer projects, and fiber coat. Nowadays, nanomaterials are considered as having likeable challenges for cleansing the small thermal conduction of operational liquids. The extensive study of thermal conductivity, nanofluid, and heat transfer can be seen in [17,18,19]. Sheikholeslami [20,21,22,23] analyzed nanofluids and their application using magnetic pitch and permeable media. Jawad et al. [24] studied Darcy–Forchheimer movement of MHD thin film nanofluid flow. Slide movement of nanoliquid film flow using graphene nanoparticles was recently studied by Khan et al. [25]. 3-D nanofluid flow with convective boundary situations is analyzed by Khan et al. [26] over a linear extending sheet.

Entropy generation is the outcome of the second law of thermodynamics [27], which states that when the system is in a stable state or undergoing reversible processes, then the amount of total entropy persists uniformly and, in irreversible (opposite) processes, the total entropy continuously increases. Irreversible processes contain the flow of fluid through a flow resistance, friction between viscid fluid and solid surface within a system, thermal resistance, diffusion, chemical reaction, and Joule heating in a fluid. Clausius [28] is the pioneer that originated the concept of entropy. Bejan [29] has initially studied the entropy generation rate and derived a method of thermodynamic optimization. Rashidi et al. [30] examined magnetohydrodynamic (MHD) flow with entropy generation in a rotating porous disk. Soomro et al. [31] inspected the numerical solution of entropy generation in MHD CNTs with water as the base fluids. Ishaq et al. [32] deliberated the thin layer nanofluid flow of nanofluids with entropy generation over an unsteady stretched surface. Some significant studies regarding entropy generation have been carried out in [33,34,35,36].

Up to now, in many different cases, the Hall current effects along with ion-slip influence are overlooked, due to their fragile role by using Ohm’s law for the weak magnitude of magnetic fields. It is well known that the abovesaid phenomena have strong effects when the power of the magnetic field is very high [37]. Generally, the Hall current effect displays an energetic character as the Hall current parameter is very high. The relation of the electron cyclotron rate to the atom electron collision rate is termed Hall parameter, where ion-slip impact is the joint impact of the flow velocities of ions and electrons [38]. Moreover, in the medical field, regarding the magnetic resonance angiography (MRA), the use of magnetic influence through the impact of Hall current, including ion-slip effects of the circulation of blood in a vein, has been most useful. MRA allows getting images of blood vessel to search for the presence of stenosis (abnormal narrowing) or any other disorders in veins of the mind, stomach, kidneys, thorax, etc. Another application is magnetic resonance imaging (MRI), which can be used to look at cancer therapy, pushing of blood, hyperthermia, and magnetic drug pointing. Motsa et al. [39] deliberated the MHD flow of micropolar liquids in the incidence of Hall current along ion-slip effect. Shah et al. [40,41] deliberated the Hall effect on micropolar nanofluid flow with radiative heat and mass transmission exploration. 

The process of heat diffusion on a rotating phenomenon indicates an important role in the industries of petrochemical, meteorology, geophysics aeronautics, and oceanography. Greenspan and Howard [42] have deliberated the rotational flow performance of viscous fluid flow and contained in a closed container with axisymmetric conditions. Nazar et al. [43] have deliberated the time-dependent behavior on rotating fluid flow and find the analytical solution of the resultant problem. Mustafa et al. [44] examined the consequence of rotational variable on a nanoliquid flow against heat transmission constant and observed that it is inversely related to it. Khan et al. [45] inspected Darcy–Forchheimer flow of micropolar nanofluid with a revolving system under the influence of non-uniform heat generation/absorption. Zin et al. [46] have inspected the rotational streaming of Jeffery nanofluid over a penetrable medium supporting the impact of nonlinear thermal emission. A numerical survey on nanofluid flow is presented by Mohammadreza et al. [47] and Hossein et al. [48]. The entropy generation of MHD nanofluid flow through a microchannel is examined by Mohammad et al. [49]. Recently, Alireza et al. [50] investigated entropy generation for MWCNT’s nanofluid flow based on water. A more detailed survey on entropy generation can be found in [51,52,53].

Lieo [54,55], in 1992, projected an analytical method named homotopy analysis method (HAM). He supposed that HAM works on the elementary idea of topology, named homotopy. For the proof of this technique, he takes two homotopic functions. When functions are continuously disintegrated to anything other than these two functions, they are called homotopic functions. This method is significant and important for solving high order nonlinear problems. By this method (HAM), we find the series solutions of a single independent variable in the form of a function which contains all the physical aspects, and we can easily discuss its behavior. The current use of HAM has been covered in [56,57] and applications of HAM can be found in [58].

The foremost purpose of discussing this work is to examine the entropy analysis of magnetohydrodynamic (MHD) nanofluid flow of single and multiwall carbon nanotubes between two revolving parallel plates. Features of both SWCNTs and MWCNTs are discussed through the Xue model. The nanofluid flow is taken under the existence of Hall current and ion-slip influence. Carbon nanotubes (CNTs) are highly proficient heat transmission agents with bordering entropy generation and, thus, are considered to be a capable cooling medium. Entropy generation and Hall effects are mainly focused upon in this work. The total entropy generation rate is calculated. Boundary-layer equations are obtained from the physical geometry. A similarity solution is obtained with the help of new variables, due to which a complicated model is transformed into simple coupled ordinary differential equations. An analytical approach is adopted for the solution of the reduced system. With the variation of different physical parameters, results are plotted, tabulated, and discussed in detail. The physical significance of Sherwood number, Nusselt number, and skin friction are present, and deliberated by graphs. Bejan number and Brinkman number effects are presented by graphs.

## 2. Basic Thermal Conductivities Models for CNTs

We discussed some basic thermal conductivity models. Maxwell [59] predicted a model of thermal conductivity for CNTs, defined as

(1) $$\frac{k_{nf}}{k_{f}}=1+\frac{3\;\left(\frac{k_{nf}-k_{f}}{k_{f}}\right)\;\varphi }{\left(\frac{k_{nf}-k_{f}}{k_{f}}+2\right)-\left(\frac{k_{nf}-k_{f}}{k_{f}}-1\right)\;\varphi }.$$

Jeffery [60] predicted it as

(2) $$\frac{k_{nf}}{k_{f}}=1+3\left(\frac{k_{nf}-k_{f}}{k_{nf}+2k_{f}}\right)\varphi +\left(3{\left(\frac{k_{nf}-k_{f}}{k_{nf}+2k_{f}}\right)}^{2}+\frac{3}{4}{\left(\frac{k_{nf}-k_{f}}{k_{nf}+2k_{f}}\right)}^{3}+\ldots \right)\;\varphi^{2}.$$

Davis [61] modified the Jeffery Model as

(3) $$\frac{k_{nf}}{k_{f}}=1+\frac{3\left(\frac{k_{nf}-k_{f}}{k_{f}}\right)\;\varphi }{\left(\frac{k_{nf}+2k_{f}}{k_{f}}\right)-\left(\frac{k_{nf}-k_{f}}{k_{f}}\right)\varphi }\;\left\{\varphi +\varphi \left(\frac{k_{nf}-k_{f}}{k_{f}}\right)\varphi^{2}+\frac{k_{nf}-k_{f}}{k_{f}}\left(\varphi^{3}\right)\right\}.$$

Hamilton and Crosser [62] proposed a new model, defined as

(4) $$\frac{k_{nf}}{k_{f}}=\frac{\frac{k_{nf}}{k_{f}}+\left(\Phi -1\right)-\left(\frac{k_{nf}-1}{k_{f}}\right)\left(\Phi -1\right)\varphi }{\frac{k_{nf}}{k_{f}}+\left(\Phi -1\right)-\left(\frac{k_{nf}-1}{k_{f}}\right)\varphi }.$$

Recently, Xue [63] projected a very important and effective model given as

(5) $$\frac{k_{nf}}{k_{f}}=\frac{1-\varphi +2\left(\frac{k_{nf}}{k_{nf}-k_{f}}\;ln\;\frac{k_{nf}+k_{f}}{2\;k_{f}}\right)\varphi }{1-\varphi +2\left(\frac{k_{f}}{k_{nf}-k_{f}}\;ln\;\frac{k_{nf}+k_{f}}{2\;k_{f}}\right)\varphi }.$$

## 3. Mathematical Modeling

We assume the flows of single and multiwall CNTs between two parallel plates. The two plates are apart from each other by a space h. Water is the base fluid. The upper plate is revolved with a continuous angular velocity γ. When γ>0, it specifies that both plates rotate in the same direction, γ<0 specifies that the plates revolve in reverse ways, γ=0 indicates that plates are in a static case. The lower plate is rotating with velocity $U_{w}=\omega x\;\left(\omega >0\right)$, quicker than the higher plates. A coordinate system (x,y,z) is nominated in a manner where the *x*-axis is corresponds to the plate, the *y*-axis is perpendicular to the plate, and the *z*-axis is perpendicular to the *xy*-plane. The plates are situated at y=0 and y=h. Applying two equal forces, but in opposite directions, kept the lower plate constant, so the position (0,0,0) cannot change. Alongside *y*, the magnetic pitch $B_{0}$ is switched, which rotates the fluid as shown in figures. Since the CNTs are electrically conducting, when the magnetic pitch is increased, Hall current yields, which distresses the CNT nanofluids. The generalized form of Ohm’s law is written as [40,41]

(6) $$J=\frac{1}{1+{\left(\frac{\omega }{v_{e}}\right)}^{2}}\left[\sigma_{nf}\left(E+\left(V\times B\right)\right)-\frac{\sigma_{nf}\left(J\times B\right)}{e\cdot \alpha_{e}}\right].$$

Here, J denotes the current density, Β is the magnetic force, Ε denotes electric intensity, ω represents electron cyclotron, $\sigma_{nf}$ denotes the nanofluid electrical conductivity, and $v_{e}$ denotes the collision frequency of electron. The law of Ohm, in view of the aforementioned circumstances, affords $\left(J_{y}=0\right)$. Using these assumptions, we obtain $J_{x}$ and $J_{y}$ [40,41]:

(7) $$J_{x}=\frac{\sigma_{nf}B_{0}^{2}}{1+m^{2}}\left(mu-w\right),$$

(8) $$J_{z}=\frac{\sigma_{nf}B_{0}^{2}}{1+m^{2}}\left(u+mw\right).$$

Here, $m=\omega_{e}t_{e}$ is the Hall constraint. The ion-slip phenomena occur when the ratio $\frac{\omega }{v_{e}}$ becomes very large.

In a revolving frame of reference, the fundamental equations for the flows are [38,39,40,41]

(9) $$\frac{\partial u}{\partial x}+\frac{\partial u}{\partial y}=0,$$

(10) $$\rho_{nf}\left(u\frac{\partial u}{\partial x}+v\frac{\partial u}{\partial y}+2\gamma w\right)=-\frac{\partial P^{*}}{\partial x}+\mu_{nf}\left(\frac{\partial^{2}u}{\partial x^{2}}+\frac{\partial^{2}u}{\partial y^{2}}\right)-\frac{\sigma_{nf}B_{0}^{2}}{\rho_{nf}\left(m_{e}^{2}+n_{e}^{2}\right)}\left(um_{e}+wn_{e}\right),$$

(11) $$\rho_{nf}\left(u\frac{\partial v}{\partial x}+v\frac{\partial v}{\partial y}\right)=-\frac{\partial P^{*}}{\partial y}+\mu_{nf}\left(\frac{\partial^{2}u}{\partial x^{2}}+\frac{\partial^{2}u}{\partial y^{2}}\right),$$

(12) $$\rho_{nf}\left(u\frac{\partial w}{\partial x}+v\frac{\partial w}{\partial y}-2\gamma u\right)=\mu_{nf}\left(\frac{\partial^{2}w}{\partial x^{2}}+\frac{\partial^{2}w}{\partial y^{2}}\right)+\frac{\sigma_{nf}B_{0}^{2}}{\rho_{nf}\left(m_{e}^{2}+n_{e}^{2}\right)}\left(un_{e}-wm_{e}\right),$$

where $P^{*}=P-\frac{\gamma^{2}x^{2}}{2},\;\sigma_{nf},\;\mu_{nf},\;m_{e}=1+n_{i}n_{e}$ represents the modified pressure, electrical conductivity, the dynamic viscosity of nanofluid, ion-slip parameter, and Hall current parameter respectively, and $P^{*}{}_{z}$ signifies the meshes cross flow alongside *z*-axis. The heat transfer equation is

(13) $$u\frac{\partial T}{\partial x}+v\frac{\partial T}{\partial y}=\frac{k_{nf}}{{\left(\rho c\right)}_{nf}}\left(\frac{\partial^{2}T}{\partial x^{2}}+\frac{\partial^{2}T}{\partial y^{2}}\right),$$

and $T,\;\alpha_{nf}=\frac{k_{nf}}{{\left(\rho c\right)}_{nf}}$ indicate the fluid temperature and thermal diffusivity respectively. The density of nanofluid and heat capacity, together with dynamic viscosity, in mathematical form, is [59,60,61,62,63]

(14) $${\left(\rho c\right)}_{nf}=\left(1-\psi \right)\rho_{f}+\psi \rho_{CNT},$$

(15) $$\rho_{nf}=\left(1-\psi \right){\left(\rho c\right)}_{f}+\psi {\left(\rho c\right)}_{CNT,\mu_{nf}}=\frac{\mu_{f}}{{\left(1-\psi \right)}^{2.5}},$$

(16) $$k_{nf}=k_{f}\left(\frac{1-\psi +2\psi \frac{k_{CNT}}{k_{CNT-k_{f}}}ln\left(\frac{k_{CNT+k_{f}}}{2k_{f}}\right)}{1-\psi +2\psi \frac{k_{f}}{k_{CNT-k_{f}}}ln\left(\frac{k_{CNT+k_{f}}}{2k_{f}}\right)}\right),$$

where $\mu_{f}$ designates the base fluid dynamic viscosity, ψ represents the nanoparticle volumetric friction, and $k_{nf}$ specify the thermal conductivity. The subscript CNT represent carbon nanotubes, f is the base fluid, and nf is the nanofluid. The boundary limitations are

(17) $$\vec{u}=U_{w}=\omega x,\;\vec{v}=0,\;\vec{w}=0,\;T=T_{h}\;\mathrm{at}\;y=0,$$

(18) $$\vec{u}=0,\;\vec{v}=-X,\;\vec{w}=0,\;T=T_{0}\;\mathrm{at}\;y=h,$$

where X indicates the constant suction/injection velocity at the high hedge. If (X), then it represents constant suction velocity and if (X), then it indicates the injection velocity. The boundary condition explained the geometry given in (Figure 1). Here, $\vec{u}=U_{w}=\omega x$ is the stretching velocity. 

Eliminating the pressure field and presenting the similarity transformations, we have

(19) $$\vec{u}=\omega xf^{\prime }\left(\xi \right),\;\vec{v}=-\omega hf\left(\xi \right),\;\vec{w}=\omega xg\left(\xi \right),\;\theta \left(\xi \right)=\frac{T-T_{0}}{T_{h}-T_{0}},\;\xi =\frac{y}{h}.$$

The non-dimensional system of equations is

(20) $$\begin{array}{l} f^{iv}\left(\xi \right)-R\left[\left(1-\psi \right)+\psi \frac{\rho_{CNT}}{\rho_{f}}\right]{\left(1-\psi \right)}^{2.5}\left(f^{\prime }\left(\xi \right)f^{\prime \prime }\left(\xi \right)-f\left(\xi \right)f^{\prime \prime \prime }\left(\xi \right)\right)\\ \;-Kr\left[\left(1-\psi \right)+\psi \frac{\rho_{CNT}}{\rho_{f}}\right]{\left(1-\psi \right)}^{2.5}g^{\prime }\left(\xi \right)\\ \;-\frac{{\left(1-\psi \right)}^{2.5}}{\mu_{f}}\left(1+\frac{3\left(\frac{\sigma_{s}}{\sigma_{f}}-1\right)\psi }{\left(\frac{\sigma_{s}}{\sigma_{f}}+2\right)-\left(\frac{\sigma_{s}}{\sigma_{f}}-1\right)\psi }\right)\frac{Μ}{\left(m_{e}^{2}+n_{e}^{2}\right)}(m_{e}f^{\prime \prime }\left(\xi \right)\\ \;+n_{e}g^{\prime }\left(\xi \right))=0, \end{array}$$

(21) $$\begin{array}{c} g^{\prime \prime }\left(\xi \right)-R\left[\left(1-\psi \right)+\psi \frac{\rho_{CNT}}{\rho_{f}}\right]{\left(1-\psi \right)}^{2.5}\left(g\left(\xi \right)f^{\prime }\left(\xi \right)-f\left(\xi \right)g^{\prime }\left(\xi \right)\right)+2Kr\left[\left(1-\psi \right)+\psi \frac{\rho_{CNT}}{\rho_{f}}\right]\\ {\left(1-\psi \right)}^{2.5}f^{\prime }\left(\xi \right)+\frac{{\left(1-\psi \right)}^{2.5}}{\mu_{f}}\left(1+\frac{3\left(\frac{\sigma_{s}}{\sigma_{f}}-1\right)\psi }{\left(\frac{\sigma_{s}}{\sigma_{f}}+2\right)-\left(\frac{\sigma_{s}}{\sigma_{f}}-1\right)\psi }\right)\frac{Μ}{\left(m_{e}^{2}+n_{e}^{2}\right)}\left(n_{e}f^{\prime }\left(\xi \right)-m_{e}g\left(\xi \right)\right)=0, \end{array}$$

(22) $$\left(\frac{1-\psi +2\psi \frac{k_{CNT}}{k_{CNT}-k_{f}}ln\frac{k_{CNT}+k_{f}}{2k_{f}}}{1-\psi +2\psi \frac{k_{f}}{k_{CNT}-k_{f}}ln\frac{k_{CNT}+k_{f}}{2k_{f}}}\right)\theta^{\prime \prime }\left(\xi \right)+R\;Pr{\left(1-\psi \right)}^{2.5}\left[\left(1-\psi \right)+\psi \frac{\rho_{CNT}}{\rho_{f}}\right]f\left(\xi \right)\theta^{\prime }\left(\xi \right)=0.$$

The relevant conditions are

(23) $$f\left(0\right)=0,f^{\prime }\left(0\right)=1,g\left(0\right)=0,\theta \left(0\right)=1,$$

(24) $$f\left(1\right)=Q,f^{\prime }\left(1\right)=0,g\left(1\right)=0,\theta \left(1\right)=0.$$

In Equation (14), $Q=\frac{X}{\omega h}$ signifies the suction/injection constrains. If (Q>0), then it represents the suction phenomenon, and if (Q<0), then it is the injection phenomenon. $R=\frac{\omega h^{2}}{\nu_{f}}\left(Reynold’s\;number\right),$ $Kr=\frac{\gamma h^{2}}{\nu_{f}}(rotation\;parameter),$ $Pr=\frac{\mu_{f}c_{p}}{k_{f}}\left(prandtl\;number\right),\;M=\frac{\sigma_{f}B_{0}^{2}h^{2}}{\rho_{f}\nu_{f}}$ is *magnetic number*.

### 3.1. Physical Quantities of Interest

The physical quantities of importance, such as skin friction and heat flux, have plentiful uses in the field of engineering. Here, we derive and discuss it. Skin friction is defined as ${\tilde{C}}_{f}=\frac{{\left(\tau_{xy}\right)}_{y=0}}{\rho_{nf}u_{w}^{2}}$. Here, $\tau_{xy}={\left({\vec{u}}_{y}\right)}^{2}$. The skin friction and Nusselt number in dimensionless form are calculated as

(25) $${\tilde{C}}_{f}=\frac{\mu_{nf}}{\mu_{f}}f^{\prime \prime }\left(0\right),$$

(26) $$Nu_{x}=-\left(\frac{k_{nf}}{k_{f}}\right)\theta^{\prime }\left(0\right).$$

### 3.2. Entropy Analysis and Bejan Numbers

The local entropy rates $S_{g.t}$ per unit volume for nanofluid according to Bejan [11,12,13,14,15,16,17,18,19,20], for the problem under attention, can be inscribed as [29,30,31,32,33,34]

(27) $$S_{g.t}^{\prime \prime \prime }=\frac{k_{nf}}{T_{0}^{2}}\left\{{\left(\nabla T\right)}^{2}\right\}+\frac{\mu_{nf}}{T_{0}}\Phi -\frac{\sigma_{nf}B_{0}^{2}}{T_{0}\rho_{nf}\left(m_{e}^{2}+n_{e}^{2}\right)}\left(um_{e}+wn_{e}\right).$$

Here, $\frac{k_{nf}}{T_{0}^{2}}\left\{{\left(\nabla T\right)}^{2}\right\}$ is the irreversibility due to heat transmission, $\frac{\mu_{nf}}{T_{0}}\Phi$ is entropy generation due to fluid friction, and $\frac{\sigma_{nf}B_{0}^{2}}{T_{0}\rho_{nf}\left(m_{e}^{2}+n_{e}^{2}\right)}\left(um_{e}+wn_{e}\right)$ signifies irreversibility due to the influence of magnetic force, where $\left(\nabla T\right)=T_{x}+T_{y}$ and Φ are related to viscous dissipation, and $\Phi ={\left({\vec{u}}_{y}\right)}^{2}.$

In our problem,

(28) $$S_{g.t}^{\prime \prime \prime }=\frac{k_{nf}}{T_{0}^{2}}\left\{{\left(\nabla T\right)}^{2}\right\}+\frac{\mu_{nf}}{T_{0}}{\left({\vec{u}}_{y}\right)}^{2}-\frac{\sigma_{nf}B_{0}^{2}}{T_{0}\rho_{nf}\left(m_{e}^{2}+n_{e}^{2}\right)}\left(um_{e}+wn_{e}\right).$$

The specific entropy rate $S_{g.c}$ is

(29) $$S_{g.c}^{\prime \prime \prime }=\frac{k_{nf}{\left(\nabla T\right)}^{2}}{T_{h}^{2}L^{2}}.$$

The entropy generation Ns is

(30) $$Ns=\frac{S_{g.t}^{\prime \prime \prime }}{S_{g.c}^{\prime \prime \prime }}.$$

Inserting Equations (28), (29), and (19) in Equation (30), we get the dimensionless form as

(31) $$Ns=Re\left[{\left(\theta^{\prime }\left(\xi \right)\right)}^{2}+\frac{1}{{\left(1-\psi \right)}^{2.5}}\frac{Br}{\lambda }\left({\left(f^{\prime \prime }\left(\xi \right)\right)}^{2}-\frac{M^{2}}{\left(m_{e}^{2}+n_{e}^{2}\right)}\left\{m_{e}{\left(f^{\prime }\left(\xi \right)\right)}^{2}+n_{e}{\left(g\left(\xi \right)\right)}^{2}\right\}\right)\right],$$

where Re,Br,λ,M are the Reynolds quantity, Brinkman quantity, non-dimensionless temperature, and Hartmann quantity, respectively, whose expressions are given by

(32) $$Re=T_{h}^{2}L^{2},\;\lambda =\frac{T_{h}-T_{0}}{T_{0}},\;Br=\frac{\mu_{f}U_{w}^{2}}{k_{nf}\left(T_{h}-T_{0}\right)}.$$

The Bejan number Be is defined as

(33) $$Be=\frac{\frac{k_{nf}}{T_{0}^{2}}{\left(T_{y}\right)}^{2}}{\frac{\mu_{nf}}{T_{0}}{\left({\vec{u}}_{y}\right)}^{2}-\frac{\sigma_{nf}B_{0}^{2}}{\rho_{nf}\left(m_{e}^{2}+n_{e}^{2}\right)}\left(um_{e}+wn_{e}\right)},$$

(34) $$Be=\frac{Re{\left(\theta^{\prime }\left(\xi \right)\right)}^{2}}{Re\left[\frac{1}{{\left(1-\psi \right)}^{2.5}}\frac{Br}{\lambda }\left\{{\left(f^{\prime \prime }\left(\xi \right)\right)}^{2}-\frac{M}{\left(m_{e}^{2}+n_{e}^{2}\right)}\left(m_{e}{\left(f^{\prime }\left(\xi \right)\right)}^{2}+n_{e}{\left(g\left(\xi \right)\right)}^{2}\right)\right\}\right]}.$$

## 4. Solution by HAM

To answer the nonlinear model and the nonlinear differential Equations (11)–(13) with boundary constraints (14), we use the homotopy analysis method (HAM). 

The initial solutions are chosen as follows:

(35) $$f\left(\xi \right)=\xi +\frac{1}{2}\left(Q-1\right)\xi^{2},f_{0}\left(\xi \right)=0,\theta_{0}\left(\xi \right)=1-\xi .$$

The linear operators are chosen as $L_{f},\;L_{\theta },\;\mathrm{and}\;L_{\varphi }$:

(36) $$L_{f}\left(f\right)=f^{\prime \prime \prime },\;L_{g}\left(g\right)=g^{\prime \prime },\;L_{\theta }\left(\theta \right)=\theta^{\prime \prime },$$

which have the general solution

(37) $$L_{f}\left(e_{1}+e_{2}\xi +e_{3}\xi^{2}\right)=0,\;L_{g}\left(e_{4}+e_{5}\xi \right)=0,\;L_{\theta }\left(e_{6}+e_{7}\xi \right)=0,$$

where $e_{i}\left(i=1-7\right)$.

The nonlinear operators $N_{f},N_{g}$, and $N_{\theta }$ are indicated as

(38) $$\begin{array}{ll} N_{f}\left[f\left(\xi ;\Phi \right),\theta \left(\xi ;\Phi \right)\right] & =\frac{\partial^{4}f\left(\xi ;\Phi \right)}{\partial \xi^{4}}\\ & -R{\left(1-\psi \right)}^{2.5}\left[\left(1-\psi \right)+\psi \frac{\rho_{CNT}}{\rho_{f}}\right]\left\{\begin{array}{c} \frac{\partial f\left(\xi ;\Phi \right)}{\partial \xi }\frac{\partial^{2}f\left(\xi ;\Phi \right)}{\partial \xi^{2}}-\\ f\left(\xi ;\Phi \right)\frac{\partial^{3}f\left(\xi ;\Phi \right)}{\partial \xi^{3}} \end{array}\right\}\\ & -Kr{\left(1-\psi \right)}^{2.5}\left[\left(1-\psi \right)+\psi \frac{\rho_{CNT}}{\rho_{f}}\right]\frac{\partial g\left(\xi ;\Phi \right)}{\partial \xi }\\ & -\frac{{\left(1-\psi \right)}^{2.5}}{\mu_{f}}\left[1+\frac{3\left(\frac{\sigma_{s}}{\sigma_{f}}-1\right)\psi }{\left(\frac{\sigma_{s}}{\sigma_{f}}+2\right)-\left(\frac{\sigma_{s}}{\sigma_{f}}-1\right)\psi }\right]\frac{M}{m_{e}^{2}+n_{e}^{2}}(m_{e}\frac{\partial^{2}f\left(\xi ;\Phi \right)}{\partial \xi^{2}}\\ & +n_{e}\frac{\partial g\left(\xi ;\Phi \right)}{\partial \xi }), \end{array}$$

(39) $$\begin{array}{ll} N_{\theta }\left[f\left(\xi ;\Phi \right),g\left(\xi ;\Phi \right)\right] & \\ & =\frac{\partial^{2}g\left(\xi ;\Phi \right)}{\partial \xi^{2}}-R{\left(1-\psi \right)}^{2.5}\left[\left(1-\psi \right)+\psi \frac{\rho_{CNT}}{\rho_{f}}\right]\left(\begin{array}{c} g\left(\xi ;\Phi \right)\frac{\partial f\left(\xi ;\Phi \right)}{\partial \xi }\\ -f\left(\xi ;\Phi \right)\frac{\partial \theta \left(\xi ;\Phi \right)}{\partial \xi } \end{array}\right)\\ & +2Kr{\left(1-\psi \right)}^{2.5}\left[\left(1-\psi \right)+\psi \frac{\rho_{CNT}}{\rho_{f}}\right]\frac{\partial f\left(\xi ;\Phi \right)}{\partial \xi }\\ & +2Kr{\left(1-\psi \right)}^{2.5}\left[\left(1-\psi \right)+\psi \frac{\rho_{CNT}}{\rho_{f}}\right]\frac{\partial f\left(\xi ;\Phi \right)}{\partial \xi }, \end{array}$$

(40) $$\begin{array}{l} N_{\theta }\left[f\left(\xi ;\Phi \right),\varphi \left(\xi ;\Phi \right)\right]\\ =Pr\left[\left(1-\psi \right)+\psi \frac{{\left(\rho c_{p}\right)}_{CNT}}{{\left(\rho c_{p}\right)}_{f}}\right]\frac{R}{\left(\frac{1-\psi +2\psi \frac{k_{CNT}}{k_{CNT}-k_{f}}ln\frac{k_{CNT}+k_{f}}{2k_{f}}}{1-\psi +2\psi \frac{k_{f}}{k_{CNT}-k_{f}}ln\frac{k_{CNT}+k_{f}}{2k_{f}}}\right)}\bar{f}\left(\xi ;\Phi \right)\frac{\partial \theta \left(\xi ;\Phi \right)}{\partial \xi }. \end{array}$$

The zero-th-order problem is

(41) $$\left(1-\Phi \right)L_{f}\left[f\left(\xi ;\Phi \right)-f_{0}\left(\xi \right)\right]=\Phi \hbar_{f}N_{f}\left[f\left(\xi ;\Phi \right),\theta \left(\xi ;\Phi \right)\right],$$

(42) $$\left(1-\Phi \right)L_{\theta }\left[\theta \left(\xi ;\Phi \right)-\theta_{0}\left(\xi \right)\right]=\Phi \hbar_{\theta }N_{\theta }\left[f\left(\xi ;\Phi \right),\theta \left(\xi ;\Phi \right)\right],$$

(43) $$\left(1-\Phi \right)L_{\varphi }\left[\varphi \left(\xi ;\Phi \right)-\varphi_{0}\left(\xi \right)\right]=\Phi \hbar_{\varphi }N_{\varphi }\left[f\left(\xi ;\Phi \right),\varphi \left(\xi ;\Phi \right)\right].$$

The correspondent boundary constrains are

(44) $${f\left(\xi ;\Phi \right)|}_{\xi =0}=0,\;{\frac{\partial f\left(\xi ;\Phi \right)}{\partial \xi }|}_{\xi =0}=1,\;{f\left(\xi ;\Phi \right)|}_{\xi =1}=Q,\;{\frac{\partial f\left(\xi ;\Phi \right)}{\partial \xi }|}_{\xi =1}=0,$$

(45) $${\theta \left(\xi ;\Phi \right)|}_{\xi =0}=0,\;{\theta \left(\xi ;\Phi \right)|}_{\xi =1}=0,\;{\varphi \left(\xi ;\Phi \right)|}_{\xi =0}=1,\;{\varphi \left(\xi ;\Phi \right)|}_{\xi =1}=0.$$

Here, Φ∈[0,1] and, in the case of Φ=0 and Φ=1, we have

(46) f(ξ;1)=f(ξ), θ(ξ;1)=θ(ξ) and φ(ξ;1)=φ(ξ).

Growing f(ξ;Φ), θ(ξ;Φ) and φ(ξ;Φ) by Taylor’s series

(47) $$f\left(\xi ;\Phi \right)=f_{0}\left(\xi \right)+\sum_{q=1}^{\infty }{\bar{f}}_{q}\left(\xi \right)\Phi^{q},$$

(48) $$\theta \left(\xi ;\Phi \right)=\theta_{0}\left(\xi \right)+\sum_{q=1}^{\infty }\theta_{q}\left(\xi \right)\Phi^{q},$$

(49) $$\varphi \left(\xi ;\Phi \right)=\varphi_{0}\left(\xi \right)+\sum_{q=1}^{\infty }\varphi_{q}\left(\xi \right)\Phi^{q}.$$

where

(50) $$f_{q}\left(\xi \right)={\frac{1}{q!}\frac{\partial f\left(\xi ;\Phi \right)}{\partial \xi }|}_{\Phi =0},\;\theta_{q}\left(\xi \right)={\frac{1}{q!}\frac{\partial \theta \left(\xi ;\Phi \right)}{\partial \xi }|}_{\Phi =0},\;\mathrm{and}\;\varphi_{q}\left(\xi \right)={\frac{1}{q!}\frac{\partial \varphi \left(\xi ;\Phi \right)}{\partial \xi }|}_{\Phi =0}.$$

As the series (50) converges at Φ=1, changing Φ=1, we get

(51) $$f\left(\xi \right)=f_{0}\left(\xi \right)+\sum_{q=1}^{\infty }f_{q}\left(\xi \right),$$

(52) $$\theta \left(\xi \right)=\theta_{0}\left(\xi \right)+\sum_{q=1}^{\infty }\theta_{q}\left(\xi \right),$$

(53) $$\varphi \left(\xi \right)=\varphi_{0}\left(\xi \right)+\sum_{q=1}^{\infty }\varphi_{q}\left(\xi \right).$$

The *q*th-order problem gratifies the following:

(54) $$L_{f}\left[f_{q}\left(\xi \right)-\chi_{q}f_{q-1}\left(\xi \right)\right]=\hbar_{f}U_{q}^{f}\left(\xi \right),$$

(55) $$L_{\theta }\left[\theta_{q}\left(\xi \right)-\chi_{q}\theta_{q-1}\left(\xi \right)\right]=\hbar_{\theta }U_{q}^{\theta }\left(\xi \right),$$

(56) $$L_{\varphi }\left[\varphi_{q}\left(\xi \right)-\chi_{q}\varphi_{q-1}\left(\xi \right)\right]=\hbar_{\varphi }U_{q}^{\varphi }\left(\xi \right).$$

The equivalent boundary conditions are

(57) $$f_{q}\left(0\right)=f_{q}^{\prime }\left(0\right)=f_{q}\left(1\right)=f_{q}^{\prime }\left(1\right)=0,$$

(58) $$\theta_{q}\left(0\right)=\theta_{q}\left(1\right)=0,\;\varphi_{q}\left(0\right)=\varphi_{q}\left(1\right)=0,$$

where

(59) $$\chi_{q}=\{\begin{array}{c} 0,\;\mathrm{if}\;\Phi \leq 1\\ 1,\;\mathrm{if}\;\Phi >1 \end{array}.$$

## 5. Results and Discussion

To determine the impact of different emerging constraints for both SWCNTs and MWCNTs based on water with Hall current effect and ion-slip consequence on velocity functions $\left(f^{\prime }\left(\xi \right)\;\mathrm{and}\;g\left(\xi \right)\right)$, temperature function g(ξ), entropy generation (Ns), and Bejan number (Be), Figure 2, Figure 3, Figure 4, Figure 5, Figure 6, Figure 7, Figure 8, Figure 9, Figure 10, Figure 11, Figure 12, Figure 13, Figure 14, Figure 15, Figure 16, Figure 17, Figure 18, Figure 19, Figure 20, Figure 21, Figure 22, Figure 23, Figure 24, Figure 25, Figure 26 and Figure 27 are plotted. These emerging parameters are nanoparticle volume friction (ψ), Reynolds number (R), rotation constraint (Kr), suction parameter (Q>0), injection parameter (Q<0), magnetic parameter (M), Prandtl number (Pr), ion-slip parameter ($n_{i}$), and Hall parameter ($n_{e}$). 

### 5.1. Velocity Profile

**Figure 2:**
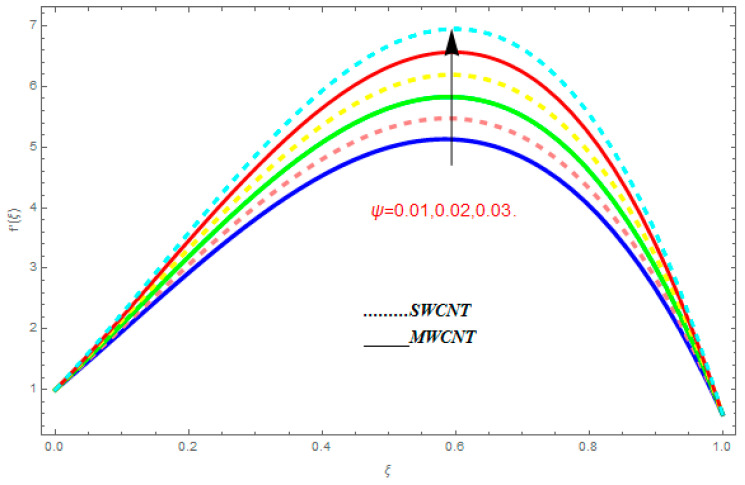
Impact of ψ on $f^{\prime }\left(\xi \right)$, when $R=0.1,\;Kr=0.5,\;n_{e}=0.6,\;Q=0.7,\;m_{e}=0.8,\;M=0.9$.

**Figure 3:**
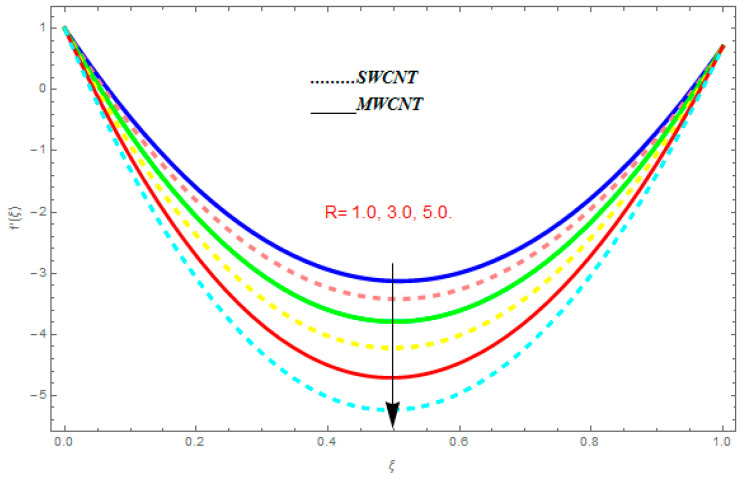
Impact of R on $f^{\prime }\left(\xi \right)$, when $\psi =0.1,\;Kr=0.5,\;n_{e}=0.6,\;Q=0.7,\;m_{e}=0.8,\;M=0.9$.

**Figure 4:**
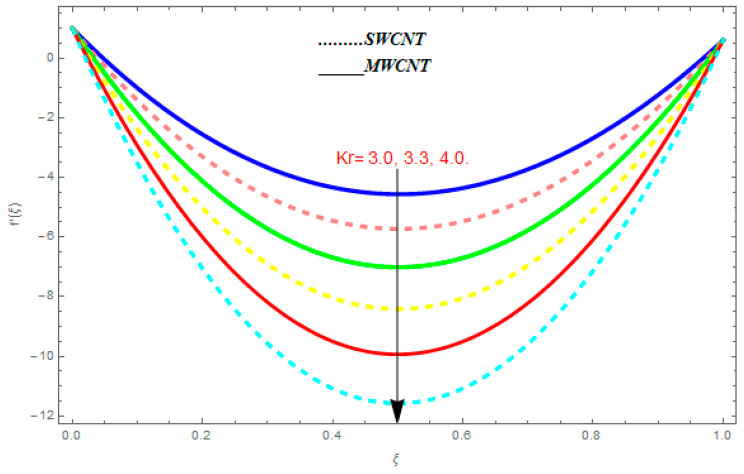
Impact of Kr on $f^{\prime }\left(\xi \right)$, when $\psi =0.1,\;R=0.5,\;n_{e}=0.6,\;Q=0.7,\;m_{e}=0.8,\;M=0.9$.

**Figure 5:**
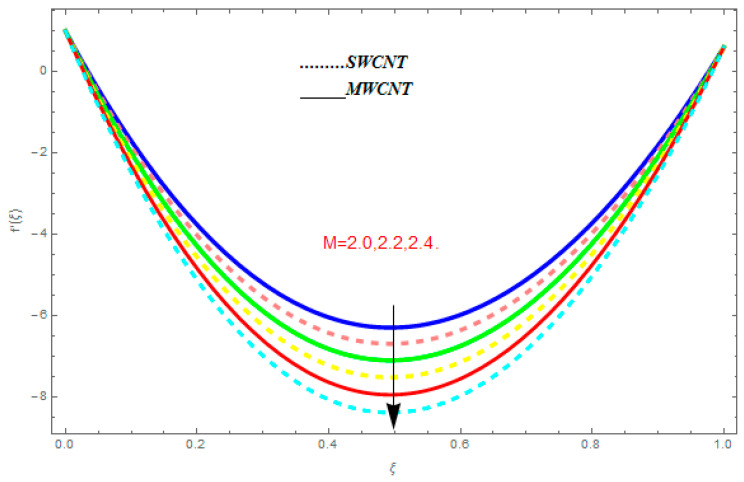
Impact of M on $f^{\prime }\left(\xi \right)$, when $\psi =0.1,\;R=0.5,\;n_{e}=0.6,\;Kr=0.7,\;m_{e}=0.8,\;Q=0.9$.

**Figure 6:**
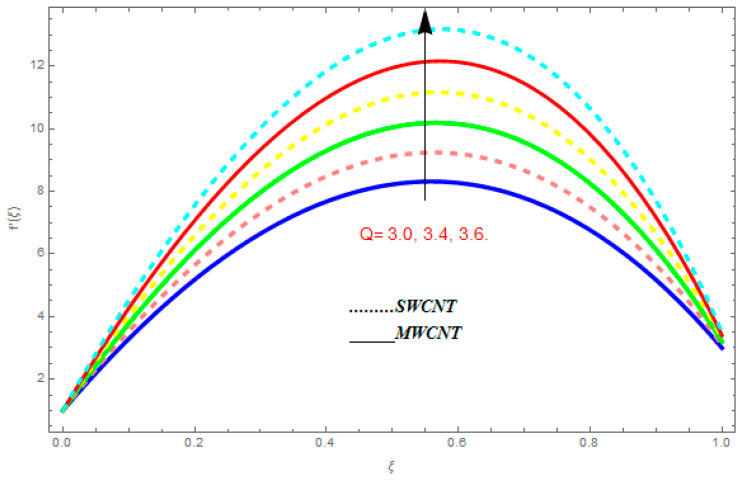
Impact of (Q>0) on $f^{\prime }\left(\xi \right)$, when $\psi =0.1,\;R=0.5,\;n_{e}=0.6,\;Kr=0.7,\;m_{e}=0.8,\;M=0.9$.

**Figure 7:**
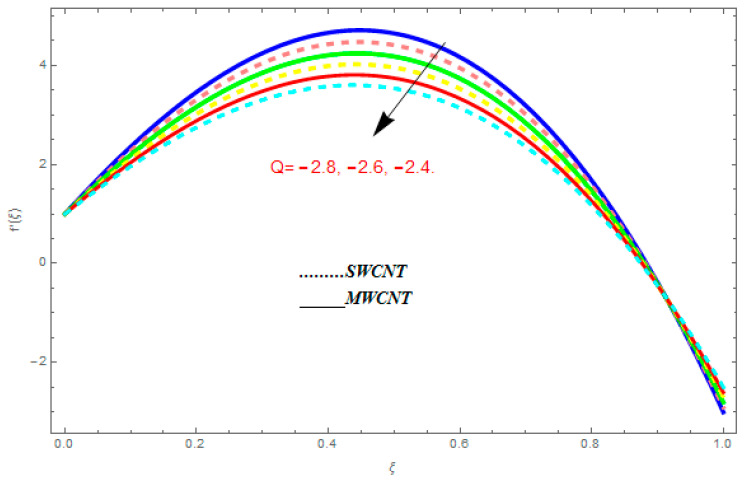
Impact of (Q<0) on $f^{\prime }\left(\xi \right)$, when $\psi =0.1,\;R=0.5,\;n_{e}=0.6,\;Kr=0.7,\;m_{e}=0.8,\;M=0.9$.

**Figure 8:**
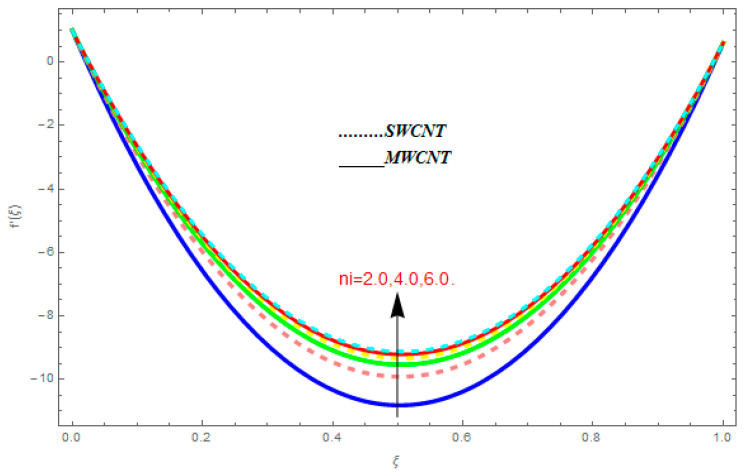
Impact of $n_{i}$ on $f^{\prime }\left(\xi \right)$, when $\psi =0.1,\;R=0.5,\;n_{e}=0.6,\;Kr=0.7,\;M=0.8,\;Q=0.9$.

**Figure 9:**
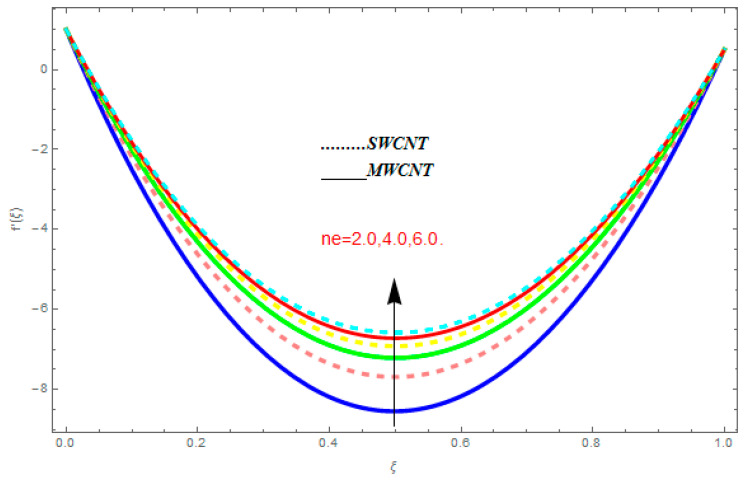
Impact of $n_{e}$ on $f^{\prime }\left(\xi \right)$, when $\psi =0.1,\;R=0.5,\;n_{i}=0.6,\;Kr=0.7,\;M=0.8,\;Q=0.9$.

**Figure 10:**
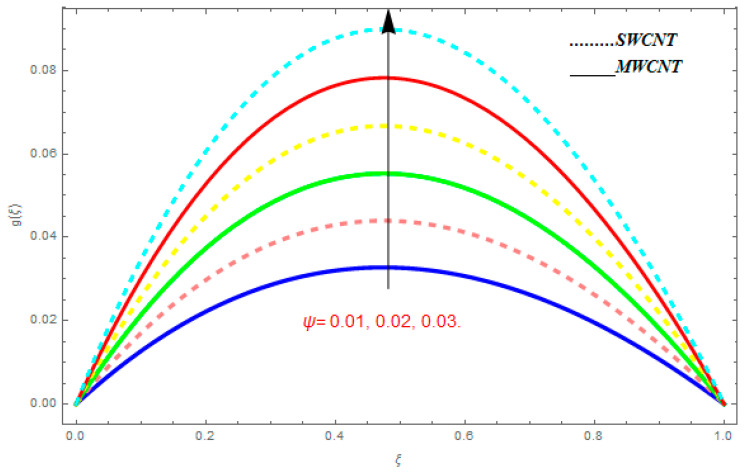
Impact of ψ on g(ξ), when $Pr=0.4,\;Kr=0.2,\;R=0.5,\;m_{e}=0.6,\;n_{e}=0.7,\;M=0.8$.

**Figure 11:**
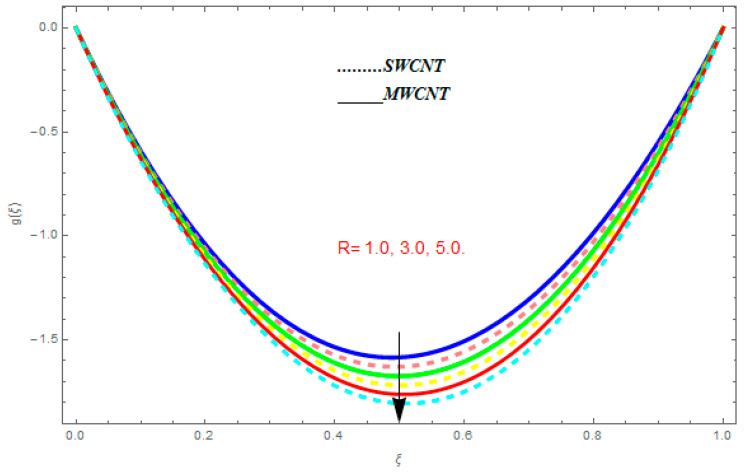
Impact of R on g(ξ), when $Pr=0.4,\;Kr=0.2,\;\psi =0.5,\;m_{e}=0.6,\;n_{e}=0.7,\;M=0.8$.

**Figure 12:**
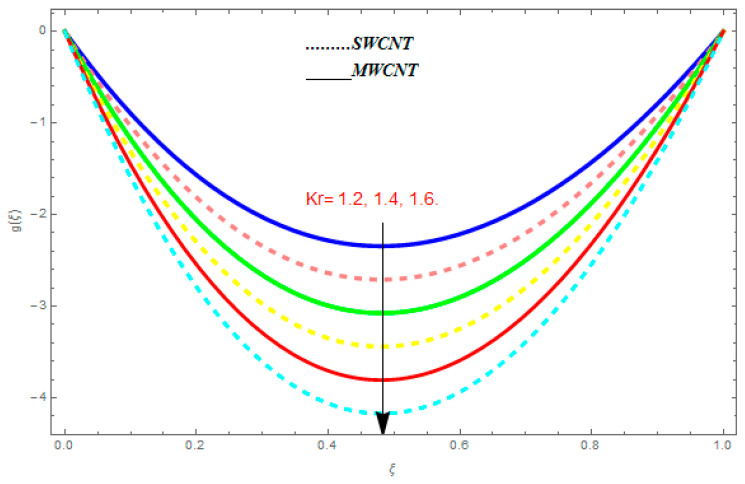
Impact of Kr on g(ξ), when $Pr=0.4,\;R=0.2,\;\psi =0.5,\;m_{e}=0.6,\;n_{e}=0.7,\;M=0.8$.

**Figure 13:**
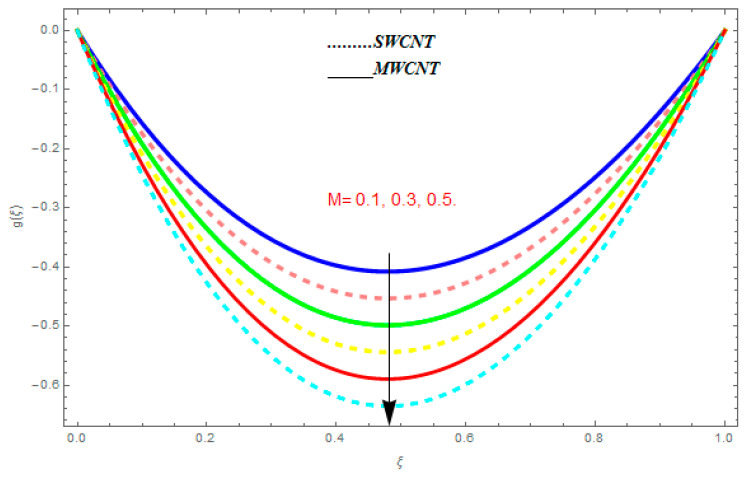
Impact of M on g(ξ), when $Pr=0.4,\;R=0.2,\;\psi =0.5,\;m_{e}=0.6,\;n_{e}=0.7,\;R=0.8$.

**Figure 14:**
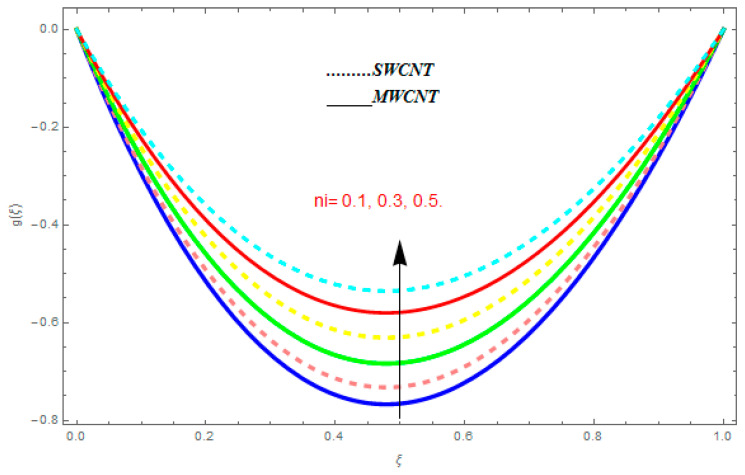
Impact of $n_{i}$ on g(ξ), when $Pr=0.4,\;R=0.2,\;\psi =0.5,\;M=0.6,\;n_{e}=0.7,\;R=0.8$.

**Figure 15:**
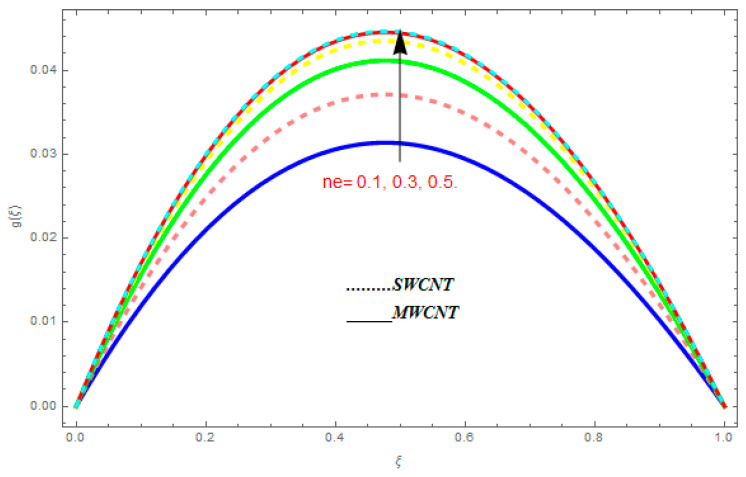
Impact of $n_{e}$ on g(ξ), when $Pr=0.4,\;R=0.2,\;\psi =0.5,\;M=0.6,\;n_{i}=0.7,\;R=0.8$.

Figure 2, Figure 3, Figure 4, Figure 5, Figure 6, Figure 7, Figure 8, Figure 9, Figure 10, Figure 11, Figure 12, Figure 13, Figure 14 and Figure 15 are designed to detect the effect of emerging constraints on $f^{\prime }\left(\xi \right)$. These constraints are nanoparticle volume friction (ψ), Reynolds quantity (R), rotation constraint (Kr), magnetic constraint (M), suction constraint (Q>0), injection constraint (Q<0), ion-slip constraint ($n_{i}$), and Hall constraint ($n_{e}$). Figure 2 is designed to realize the association between the SWCNTs and MWCNTs with the rising values of volume fraction ψ of nanoparticle. It is clear from the figure that there is an inconsequential difference in $f^{\prime }\left(\xi \right)$ with the higher values of ψ. Actually, Reynolds number is the main effect on the behavior of the flow. It is determined that the MWCNTs have relatively much better $f^{\prime }\left(\xi \right)$ as related to SWCNTs. Figure 3 shows attempts to understand the influence of R on $f^{\prime }\left(\xi \right)$. It is one of the most significant phenomena of particles in a fluid. It reduces the boundary layer thickness with higher values of R. Therefore, from the figure, R shows a reduction in $f^{\prime }\left(\xi \right)$. Figure 4 shows the impact of Kr on $f^{\prime }\left(\xi \right)$. Physically, the rotation reduces its linear velocity, resulting in the rotation and a uniform flow of the liquid. In the case of a large magnitude of Kr, resistance is provided to the fluid flow. Therefore, the velocity function $f^{\prime }\left(\xi \right)$ reduces with augmented values of Kr. In addition, due to the high density of SWCNTs, the velocity function of SWCNTs is lower in magnitude, as is the case with MWCNTs. Figure 5 is plotted to see the effect of M on $f^{\prime }\left(\xi \right)$. According to Lorentz force theory, the magnetic pitch constraint has an opposite result on $f^{\prime }\left(\xi \right)$, that is, $f^{\prime }\left(\xi \right)$ reduces with the augmentation in M. This impact of magnetic field M on velocity pitch, is the reason that the growth in magnetic field M develops a friction force, called the Lorentz force. This has the attraction of being able to diminish the velocity pitch at the boundary surface, where an alternative force known as the Carioles force shows the opposite impact on velocity. Figure 6 displays the impact of (Q>0) on $f^{\prime }\left(\xi \right)$. From the figure, we see that $f^{\prime }\left(\xi \right)$ increases with suction (Q>0), that is, $f^{\prime }\left(\xi \right)$ rises with the positive values of Q. However, from Figure 7, it can be detected that $f^{\prime }\left(\xi \right)$ reduces with injection (Q<0), that is, $f^{\prime }\left(\xi \right)$ diminishes with the negative value of Q. It is properly observable that the occurrence of CNTs nanoparticles have better $f^{\prime }\left(\xi \right)$ function, while $f^{\prime }\left(\xi \right)$ function rises more when (Q>0) exists. However, the declarations in $f^{\prime }\left(\xi \right)$ function are due to the fact that (Q<0) absorbs the internal heat energy from the surface. Figure 8 is plotted to see the effect of $n_{i}$ on $f^{\prime }\left(\xi \right)$. It is clear from the figure that higher values of $n_{i}$ show an escalation in $f^{\prime }\left(\xi \right)$. Generally, in addition to ion-slip parameter, the velocity function accelerates and, as a result, the thickness of the boundary stream improves. Therefore, the velocity fields accelerate with the increasing values of ion-slip parameter. Figure 9 shows the impact of $n_{e}$ on $f^{\prime }\left(\xi \right)$. It is indicated from the figure that a growing number of $n_{e}$ shows a reduction in $f^{\prime }\left(\xi \right)$. The existence of $n_{e}$ reduces the opposing force accomplished by the magnetic field. Hence, $f^{\prime }\left(\xi \right)$ increases with the escalation in $\;n_{e}$. In addition, it is clear from Figure 8 and Figure 9 that $n_{i}$ is more effective on $f^{\prime }\left(\xi \right)$ than $n_{e}$. Figure 2, Figure 3, Figure 4, Figure 5, Figure 6, Figure 7, Figure 8 and Figure 9 show attempts to see the effect of emerging limitations on g(ξ). These constraints are nanoparticle volume friction (ψ), Reynolds number (R), Rotation constraint (Kr), Magnetic constraint (M), ion-slip constraint ($n_{i}$), and Hall constraint ($n_{e}$). Figure 10 shows an attempt to understand the association between the SWCNTs and MWCNTs with the growing values of volume fraction ψ of nanoparticles. From Figure 10, plenty of disparity is observed between g(ξ) and the growing values of ψ. We also detected that the MWCNTs has relatively much better g(ξ) as related to SWCNTs. Figure 11 is an attempt to understand the influence of R on g(ξ). It is one of the most significant phenomena of particles in a fluid. It reduces the boundary layer thickness with the escalating values of R. Therefore, from the Figure 11, R shows a reduction in g(ξ). Figure 12 shows the impact of Kr on g(ξ). For large values of Kr, a large resistance has been provided to the fluid flow. Therefore, the velocity function g(ξ) reduces with escalating values of Kr. In addition, due to high density of SWCNTs, the velocity function of SWCNTs is lower in magnitude, as of MWCNTs. Figure 13 is plotted to see the effect of M on g(ξ). According to Lorentz force theory, the magnetic pitch constraint has a converse consequence on g(ξ), that is, θ(ξ) reduces with the augmentation in M. Figure 14 is designed to understand the consequence of $n_{i}$ on g(ξ). It is clear from the figure that the growing values of $n_{i}$ lead to an escalation in g(ξ). Generally, in addition to ion-slip parameter, the velocity function accelerates and, as a result, the thickness of the boundary stream improves. Therefore, the velocity function accelerates with the cumulative values of ion-slip constraint. Figure 15 shows the impact of $n_{e}$ on g(ξ). It is clear from the figure that higher values of $n_{e}$ show a reduction in g(ξ). The existence of $n_{e}$ reduces the opposing force accomplished by magnetic field. Hence, g(ξ) increases with the escalation in $n_{e}$. In addition, it is clear from Figure 14 and Figure 15 that $n_{i}$ is more effective on θ(ξ) than $n_{e}$.

### 5.2. Temperature Function θ(ξ)

**Figure 16:**
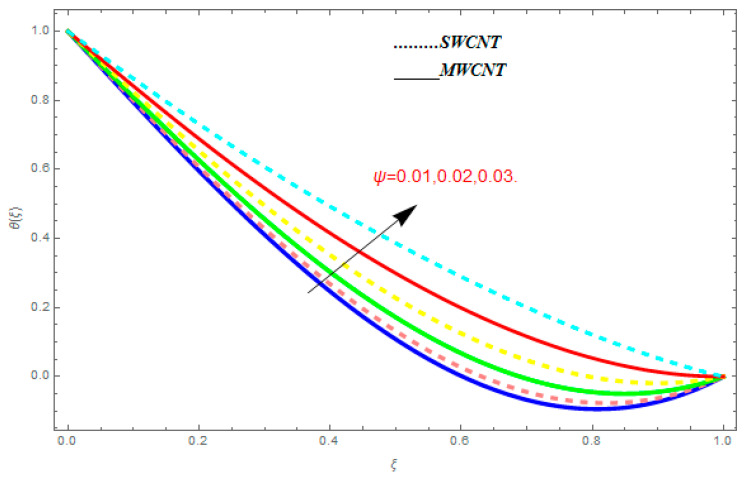
Impact of ψ on θ(ξ), when Pr=0.9, R=0.1.

**Figure 17:**
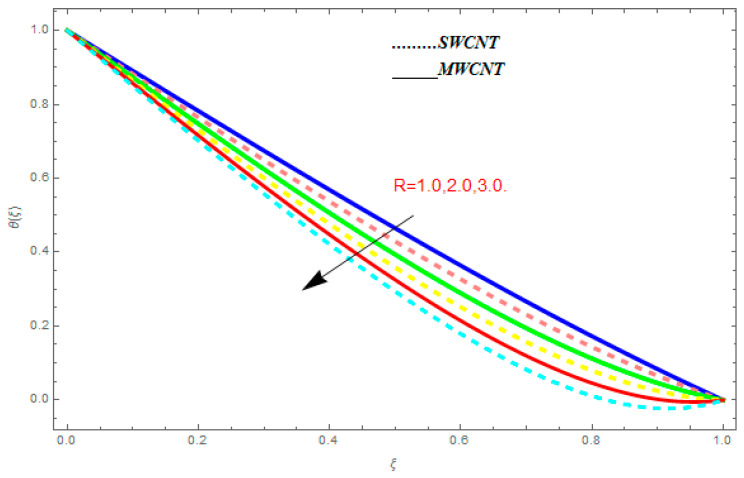
Impact of R on θ(ξ), when ψ=0.1, Pr=0.9.

**Figure 18:**
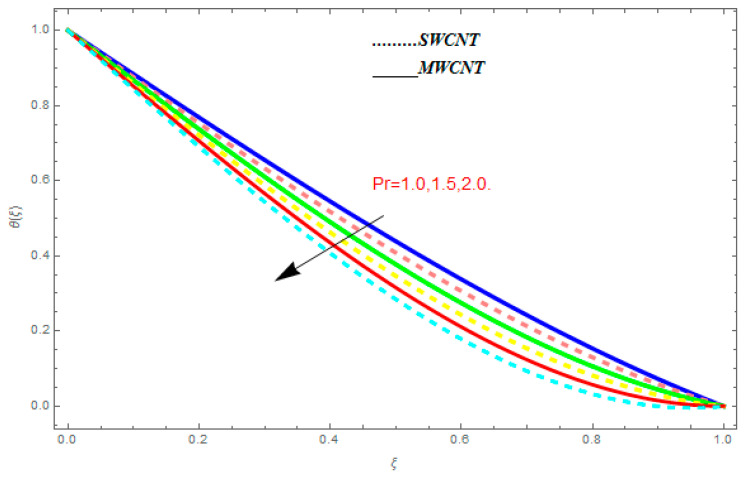
Impact of r on θ(ξ), when ψ=0.1, R=1.0.

Figure 16, Figure 17 and Figure 18 are plotted to see the effect of emerging parameters on θ(ξ). These constraints are nanoparticle volume friction (ψ), Reynolds number (R), and Prandtl number (Pr). Figure 16 shows attempts to understand the association between the SWCNTs and MWCNTs through the growing numbers of volume fraction ψ of nanoparticles. From Figure 16, plenty of disparity is observed between θ(ξ) and the growing numbers of ψ. We noticed that the MWCNTs have relatively much superior θ(ξ) as associated to SWCNTs. Figure 17 is an attempt to understand the influence of R on θ(ξ). It is one of the most significant phenomena of particles in fluid dynamics. Higher values of R reduce the boundary layer thickness with higher values of R. Figure 18 is plotted to see the impact of Pr. Prandtl number represents the ratio of specific heat capacity and dynamic viscosity with the thermal conductivity of the primary fluid. Higher Prandtl number possesses a greater thermal conductivity and this results in a significant decrease in the temperature function θ(ξ). Therefore, the escalating values of Pr show decreasing behavior in temperature function θ(ξ). Physically, the fluids with a small amount of Pr have greater thermal diffusion and, as a result, are reversed for greater Prandtl number.

### 5.3. Entropy Generation (Ns) and Bejan Number (Be)

**Figure 19:**
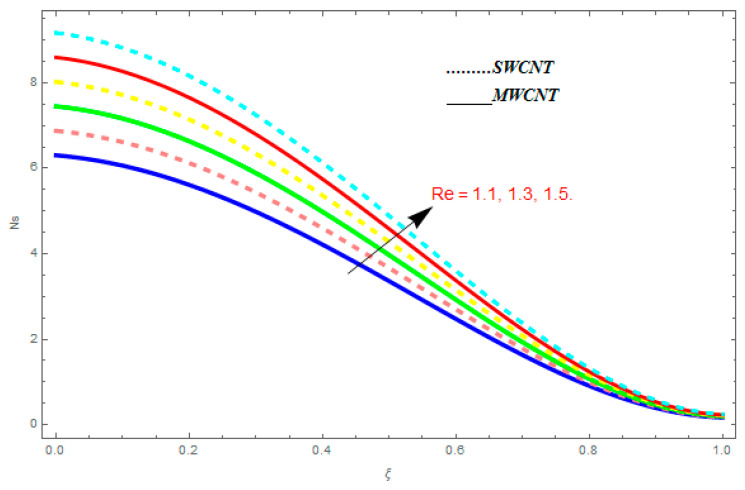
Impact of Re on Ns, when $M=0.1,\;Br=0.5,\;n_{e}=0.4,\;n_{i}=0.6$.

**Figure 20:**
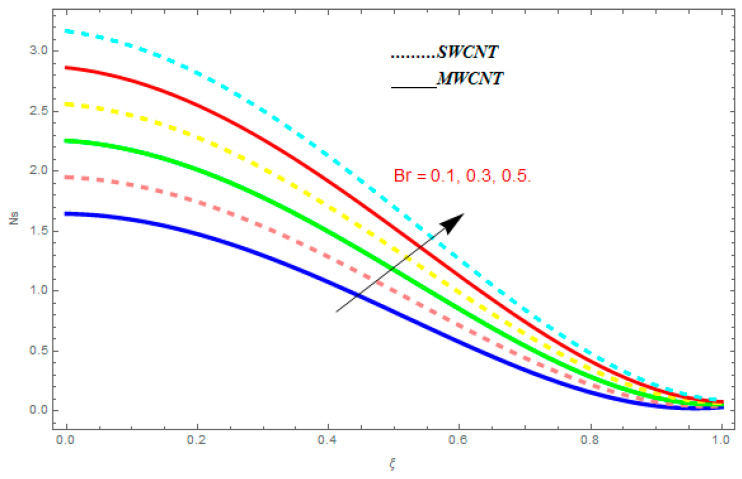
Impact of Br on Ns, when $Re=0.5,\;M=0.1,\;n_{e}=0.4,\;n_{i}=0.6$.

**Figure 21:**
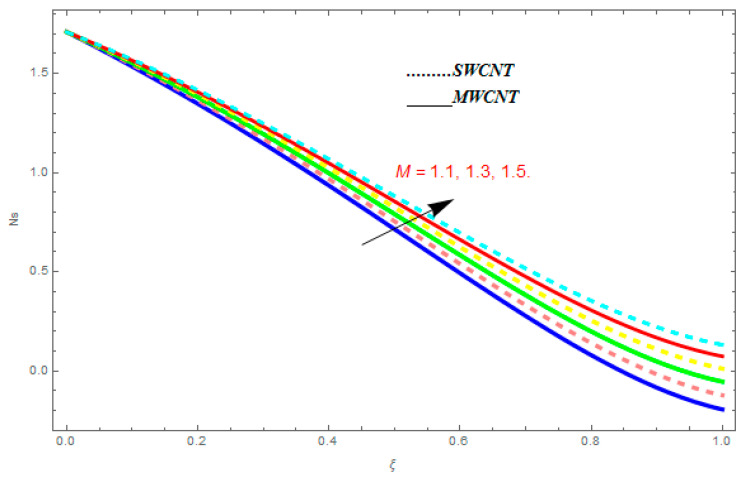
Impact of M on Ns, when $Re=0.5,\;Br=0.1,\;n_{e}=0.4,\;n_{i}=0.6$.

**Figure 22:**
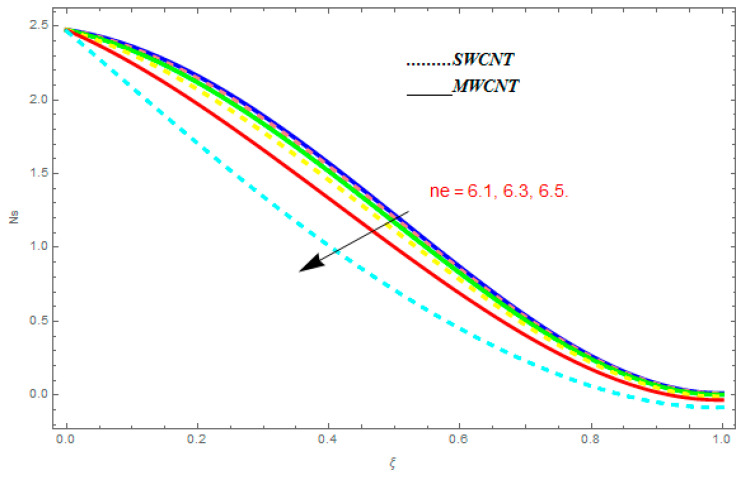
Impact of $n_{e}$ on Ns, when $Re=0.5,\;Br=0.1,\;M=0.4,\;n_{i}=0.6$.

**Figure 23:**
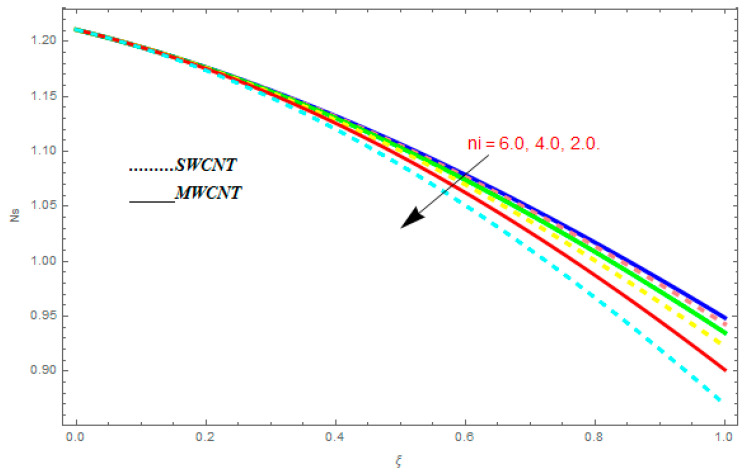
Impact of $n_{i}$ on Ns, when $Re=0.5,\;Br=0.1,\;M=0.6,\;n_{e}=0.6$.

In this section, we deliberated the entropy generation and the impact of physical parameter on it. Figure 19, Figure 20, Figure 21, Figure 22 and Figure 23 show the influences of emerging parameters on entropy generation Ns. These parameters are Reynolds number (Re), Brinkman number (Br), magnetic constraint (M), Hall constraint ($n_{e}$), and ion-slip constraint ($n_{i}$). Generally, the entropy generation Ns is found to be higher for SWCNTs as compared to MWCNTs. Figure 19 shows the impact Re on Ns. It is clear from the figure that the growing values of Re shows reducing performance in Ns. The same result is found for both SWCNTs and MWCNTs. Figure 20 shows the influence of Br on Ns. It is noticed that when Br is increased, entropy generation increases. In addition, an increase in Ns, created by fluid friction and joule dissipation, arises through the higher values of Br. The characteristic of magnetic parameter M on entropy profile is shown in Figure 21. From the figure, we observed that the augmented values of M raise the behavior of entropy profile Ns. The characteristic of Hall parameter and ion-slip parameter are shown in Figure 22 and Figure 23. From these graphs, we observed that greater values of Hall constraint and ion-slip constraint reduce the entropy generation. 

**Figure 24:**
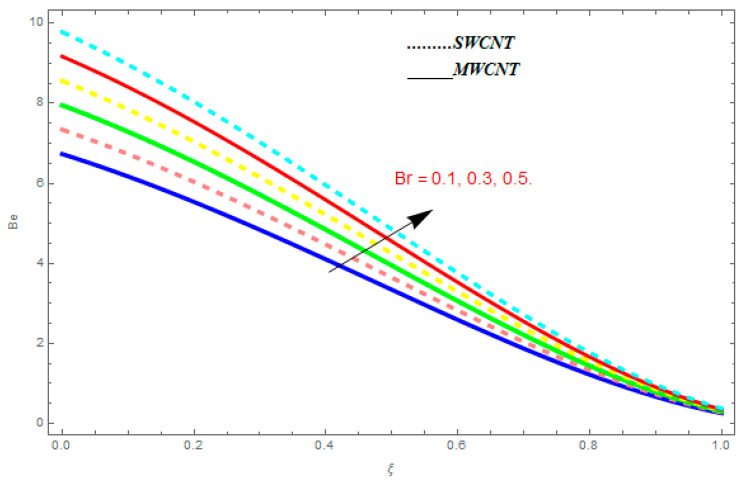
Impact of Br on Be, when $M=0.1,\;n_{e}=0.4,\;n_{i}=0.6$.

**Figure 25:**
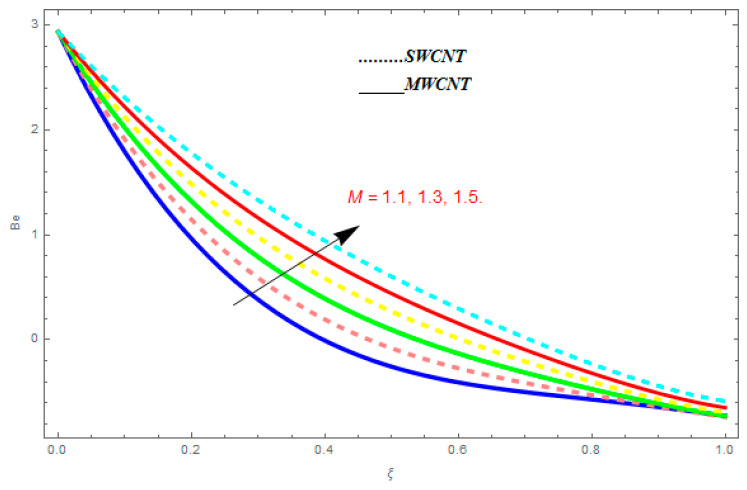
Impact of M on Be, when $Br=0.1,\;n_{e}=0.4,\;n_{i}=0.6$.

**Figure 26:**
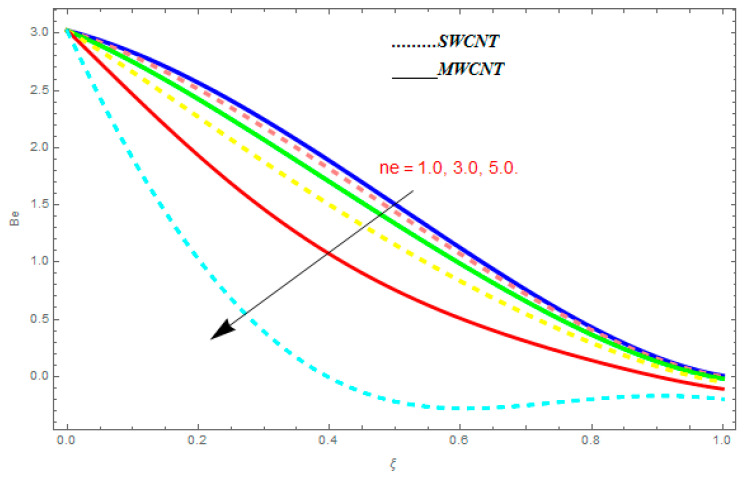
Impact of $n_{e}$ on Be, when $Br=0.1,\;M=0.4,\;n_{i}=0.6$.

**Figure 27:**
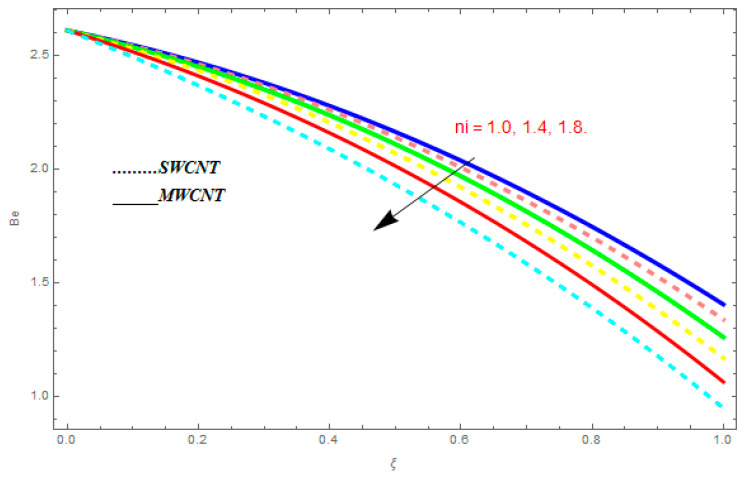
Impact of $n_{i}$ on Be, when $Br=0.1,\;M=0.4,\;n_{e}=0.6$.

Figure 24, Figure 25, Figure 26 and Figure 27 are plotted to understand the impacts of emerging constraints on entropy generation Be. These parameters are Brinkman number (Br), magnetic constraint (M), Hall parameter ($n_{e}$), and ion-slip parameter ($n_{i}$). Figure 24 shows the influence of Br on Be. An increase in Br shows escalating behavior in Be. Figure 25 shows the influence of M on Be. It is perfect from the figures that the growing values of M show increasing behavior in Be. Figure 26 and Figure 27 show the impact of $n_{e}$ and $n_{i}$ on Be. From these figures, we understand the growing values of $n_{e}$ and $n_{i}$ show diminishing behavior in Be.

### 5.4. Tables Discussion 

Table 1 and Table 2 show attempts to understand the impacts of emerging parameters for both SWCNTs and MWCNTs–kerosene oil with Hall current effect and ion-slip effect on skin friction factor ${\tilde{C}}_{f}$ and local Nusselt number $Nu_{x}$. These emerging parameters are Reynolds quantity (R), rotation constraint (Kr), magnetic constraint (M), suction constraint (Q>0), injection constraint (Q<0), ion-slip constraint ($n_{i}$), Hall constraint ($n_{e}$), and Prandtl number (Pr). Table 1 shows the impact of emerging parameters on ${\tilde{C}}_{f}$. We see from the table that with increasing values of Kr, M, and (Q<0), the skin friction coefficient is reduced while, with growing numbers of R, (Q>0), $n_{i}$, and $n_{e}$, the skin friction coefficient, escalate. Table 2 shows the impact of different emerging parameters on $Nu_{x}$. We see from the table that with the escalating values of R, Kr, $n_{e}$, M, and (Q<0), the local Nusselt number is reduced and, with (Q>0) and $n_{e}$, the local Nusselt number escalates while the Prandtl number (Pr) was not affected by the local Nusselt number. Table 3 is planned to revise the physical belongings of CNTs, thermophysical properties CNTs and nanofluids of certain base fluids, and thermal conduction ($k_{nf}$) of CNTs with dissimilar volume friction (φ), correspondingly.

## 6. Conclusions

Entropy generation investigation for the three-dimensional rotating flow with Hall current and ion-slip properties of a CNT nanofluid over a sloped stretched surface with suction/injection, MHD, and radiative heat flux properties, were inspected. The model partial differential equations (PDEs) are converted to nonlinear ordinary differential equation (ODEs) by using with the help of similarity variables. The influences of inserted of constraints were shown graphically. Upon the completion of study, important comments and conclusions which were reached are outlined below.

(a) The velocity function $f^{\prime }\left(\xi \right)$ increased with the augmentation in φ, positive Q, $n_{i}$, and $n_{e}$, while it reduced with higher values of R, M, Kr, and negative Q.
(b) It is observed that the transverse velocity function g(ξ) increased with greater value of $\varphi ,\;n_{i},\;\mathrm{and}\;n_{e}$, while it showed a reducing behavior for higher values of R, Kr, and M.
(c) The temperature function θ(ξ) was augmented with the augmentation in φ, while it showed reducing behavior with the escalation in R, Pr.
(d) For entropy profile, it was observed that entropy generation Ns increased with higher value of M, Re, and Br, while it showed decreasing behavior with an increase in $n_{i}\;\mathrm{and}\;n_{e}$.
(e) The Bejan number Be showed increasing behavior with an increase in M, Br, while it showed decreasing behavior with an increase in $n_{i}\;\mathrm{and}\;n_{e}$.

## Figures and Tables

**Figure 1:**
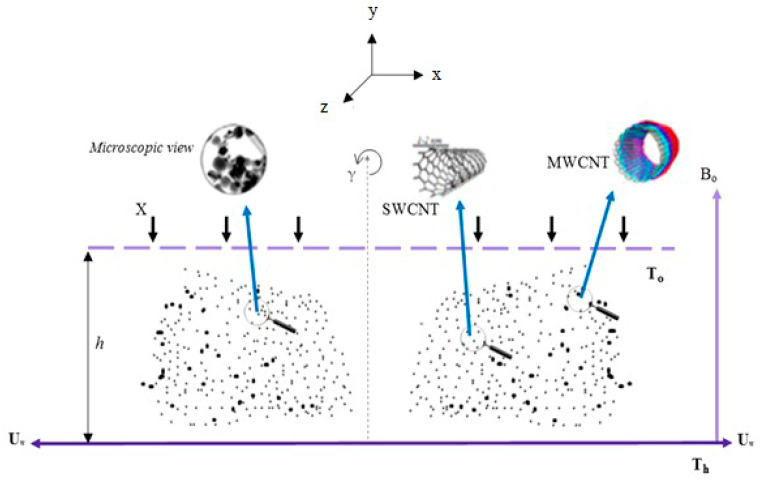
Geometry of the state problem.

**Table 1:** The numerical values of skin friction ${\tilde{C}}_{f}=\frac{\mu_{nf}}{\mu_{f}}f^{\prime \prime }\left(0\right),$ when ψ=0.01.

R	Kr	M	Q	$n_{e}$	$n_{i}$	${\tilde{C}}_{f}$ at ξ=0	${\tilde{C}}_{f}$ at ξ=1
0.1	0.2	0.4	0.5	0.3	0.2	−0.484638	−0.515879
0.3						−0.470976	−0.529161
0.5	0.2					−0.458474	−0.541466
	0.4					−0.461839	−0.538401
	0.6	0.4				−0.465886	−0.534719
		0.7				−0.472578	−0.529764
		1.0	−1.5			−0.483196	−0.521086
			−0.1			−2.187070	−1.202060
			0.1			−1.006670	−0.971885
			1.5	0.3		−0.835078	0.523539
				0.4		0.491335	0.511560
				0.5	0.2	0.498996	−0.503849
					0.6	0.490763	0.511484
					1.0	0.495080	0.507058

**Table 2:** The numerical values of Nusselt number $Nu_{x}=-\left(\frac{k_{nf}}{k_{f}}\right)\varphi^{\prime }\left(0\right),$ when ψ=0.01.

R	Kr	M	Q	$n_{e}$	$n_{i}$	Pr	$Nu_{x}$ at ξ=0	$Nu_{x}$ at ξ=1
0.1	0.2	0.4	0.5	0.3	0.2	7.2	−0.001105	0.000884
0.3							−0.001107	0.000886
0.5	0.2						−0.001110	0.000889
	0.4						−0.001445	0.001157
	0.6	0.4					−0.001779	0.001429
		0.7					−0.002337	0.001887
		1.0	−1.5				−0.002932	0.002347
			−0.1				−0.000808	0.000995
			0.1				−0.002280	0.001443
			1.5	0.3			−0.002596	0.004649
				0.4			−0.003721	0.004249
				0.5	0.2		−0.003404	0.003887
					0.6		−0.002348	0.002682
					1.0	7.2	−0.001862	0.002126
						7.3	−0.001862	0.002126
						7.5	−0.001862	0.002126

**Table 3:** Physical properties of carbon nanotubes (CNTs) (Xie et al. [50]).

Materials	SWCNTs	MWCNTs
**Thermal Conductivity** $k_{nf}$ **(**W/mK**)**	3000	3000
**Specific gravity** **(g/cm^3^)**	0.8	1.8
**Strength** (GPa)	50–500	10–60
**Elastic Modulus** (TPa)	1	0.3–1

## Nomenclature

*Pr*	Prandtl number (=υ/α)	ψ	similarity variables
*P*	fluid pressure (Pa)	$\tau_{xy}$	surface shear stress
Nu	Nusselt number	σ	electrical conductivity
g	internal energy distribution functions	α	thermal diffusivity
$B_{0}$	magnetic flux density $\left({\mathrm{NmA}}^{-1}\right)$	*τ*	lattice relaxation time
Be	Bejan number.	φ	volume friction
m	Hall parameter	k	thermal conductivity
M	magnetic parameter	α	thermal diffusivity $\left(m^{2}s^{-1}\right)$
J	current density	Ω	angular velocity $\left({\mathrm{ms}}^{-1}\right)$
E	electric intensity	μ	dynamic viscosity (mPa)
$n_{e}$	ion-slip parameter	ω	electron cyclotron
*a*, *b*, *c*	constants		
T	fluid temperature K	***Subscripts***	
$c_{p}$	specific heat $\left(\frac{J}{kgK}\right)$	nf	nanofluid
		CNTs	carbon nanotubes
${\tilde{C}}_{f}$	skin friction coefficient	h	hot
R	Reynolds number	ave	average
Q	suction and injection		
Kr	rotation parameter		
h	distance between the plates (m)		
$Q_{w}$	surface heat flux		
$S_{h},S_{R},S_{f}$	Dimensional entropy generation		
Ns	non-dimensional entropy generation		
u, v w	velocities components $\left({\mathrm{ms}}^{-1}\right)$		
x,y,z	coordinates		
O	origin		
$Nu_{x}$	Nusselt number		
***Greek symbols***			
υ	kinematic viscosity		
ρ	fluid density		
